# Production of an ultrasound-assisted biosurfactant postbiotic from agro-industrial wastes and its activity against Newcastle virus

**DOI:** 10.3389/fnut.2022.966338

**Published:** 2022-09-26

**Authors:** Asma Behzadnia, Marzieh Moosavi-Nasab, Ali Mohammadi, Siavash Babajafari, Brijesh K. Tiwari

**Affiliations:** ^1^Department of Food Science and Technology, School of Agriculture, Shiraz University, Shiraz, Iran; ^2^Seafood Processing Research Centre, School of Agriculture, Shiraz University, Shiraz, Iran; ^3^Nutrition Research Center, Shiraz University of Medical Sciences, Shiraz, Iran; ^4^Department of Pathobiology, School of Veterinary Medicine, Shiraz University, Shiraz, Iran; ^5^Department of Clinical Nutrition, Nutrition Research Center, School of Nutrition and Food Sciences, Shiraz University of Medical Sciences, Shiraz, Iran; ^6^Department of Food Chemistry and Technology, Teagasc Food Research Centre, Dublin, Ireland

**Keywords:** agro-industrial wastes, biosurfactant, *Lactobacillus plantarum*, central composite design, ultrasonication, antiviral activity

## Abstract

The objective of this study is to optimize the biosurfactant production by *Lactobacillus plantarum* ATCC 8014 using low-cost substrates from industrial sources applying ultrasonication at 28 kHz frequency (power of 100 W). Given this, whey permeate and sugar cane molasses were screened to continue optimization using a central composite design to improve the production. Then, the effect of ultrasound was examined at different stages of microbial growth. The combination of whey permeate and sugar cane molasses with yeast extract (2.4 g/L) and inoculum size of 4.8% for 26 h of fermentation time significantly influenced biosurfactant production by reducing the surface tension of water (41.86 ± 0.24 mN/m). Moreover, ultrasonication led to the further reduction in surface tension value (39.95 ± 0.35 mN/m). Further, no significant differences were observed between products from synthetic and waste-based media. The biosurfactants exhibited antiviral activity against Newcastle disease virus (NDV) LaSota strain. It was discovered that biosurfactant produced in agro-food wastes with a significant antiviral effectiveness could be used to develop commercial application instead of chemical surfactants and biosurfactants from expensive synthetic media.

## Introduction

Lactic acid bacteria synthesize different bioactive metabolites including bacteriocins, biosurfactants, short-chain fatty acids, organic acids, enzymes, carbohydrates, alcohols vitamins, cofactors, immune-signaling compounds and low molecular mass substances. The antimicrobial substances produced by lactic acid bacteria are known as postbiotics and have determined extremely important to inhibit pathogenic organisms and improve the food safety and the health of the consumers (1, 2). Therefore, postbiotics are recognized as microbial metabolites from food-grade microorganisms during fermentation process cell-bound or excreted to the culture media, in food or gut including some benefits to the food or hosts (1). Biosurfactants recently known as postbiotics with antimicrobial activities and several health benefits to the consumers. The use of microorganisms (e.g., bacteria, yeasts, and filamentous fungi) is outperforming the chemical processes to produce surfactants. Thus, the production of biosurfactants has been provoked enhanced scientific attention in the field of bio-surfactants (3, 4). Biosurfactants provide competitive advantages due to their chemical composition and structure including (i) low toxicity, (ii) high biodegradability, and (iii) effectiveness under intensive physical conditions in terms of pH, temperature, and salinity; (iv) low critical micelle concentration (CMC); and (v) better surface activity in comparison to their chemically-synthesized counterparts (3, 5). As the surface tension value is the main parameter indicating biosurfactant production, decrease on the surface tension of the media (such as water) demonstrates the effectiveness and efficiency of the produced biosurfactant, however, the lower CMC means that less surfactant is necessary to get a maximal decrease on surface tension (6). Moreover, biosurfactants have a variety of properties such as emulsifiers, demulsifiers, foaming agents, detergents and also pore formation capability and cell membrane instabilization, which can be utilized as antibacterial, antifungal, antiviral and anti-adhesive agents against a range of foodborne pathogens (7, 8). These particular properties alongside their ecologically friendly nature lead to biosurfactants potential constituents with extensive utilizations in the pharmaceutical, biomedical, food, cosmetic, oil, and petroleum, biodegradation and pesticide industries (9).

Despite the several benefits of assembling microbial biosurfactants, the commercialization is hampered due to the fact that fermentation processes have a low yield, which makes them expensive to produce, not being able to economically compete with synthetic surfactants (3). Expensive raw materials constitute 10–30% of the total cost of large-scale bioprocesses, are the main concern for biosurfactant production (10, 11). One strategy into making biosurfactants production more cost-effective is the replacement of synthetic substrates by agro-food wastes and renewable substrates representing an additional advantage as well as a decrease in environmental concern while it provides a high-product yield (11–13). The correct balance of nutrients is an important factor to select the right biowastes for microbial growth and production of metabolites like biosurfactants (10, 14). Currently, a wide range of renewable raw substrates like various industrial and agricultural by-products and wastes are available that can be employed as nitrogen and carbon sources as well as other nutrient minerals for industrial fermentation processes (10, 12). Research studies have demonstrated the use of alternative substrates for the production of biosurfactants. These include molasses, whey, and cassava flour wastewater (15, 16), beet pulp and potato peels (17), coffee wastewater (11), low-quality date syrup (18), soybean frying oil waste and corn steep liquor (19). Nevertheless, the complexity of such agro-industrial substrates restricts the bioavailability of nutrients that needs to be broken down to conversion into smaller forms (20). As well as the noticed limitations facing industrial biosurfactant production, the slow kinetics of fermentation processes play a crucial role in the productivity and the final process prices. In addition to the alternative substrates applied to the biosurfactant production, the potential of emerging non-thermal technologies such as ultrasound technique would be an effective approach to improve the efficiency of the biosurfactants production, while there are not yet vast reported studies (21, 22).

Power ultrasound is a versatile technology that has been known as the technique of extraction, emulsification, degassing, cutting, and process monitoring in a range of industries, including food, fine chemicals, medical, biotechnological, and pharmaceutical (22, 23). Power ultrasound has also been demonstrated to induce injures of varying degree to the microorganisms (23, 24). However, the application of ultrasonication is a relatively new approach to complex stimulate living organisms *via* enhancing membrane penetration and transportation through the cells, shortening the microbial lag phase, accelerating the microbial cell growth and microbial metabolism leading to lucrative productivities (20, 22, 25). Formation of the acoustic cavitation during ultrasonication creates repairable holes on the cell membrane, or even influences the cellular metabolism which may facilitate nutrient consumption, and intracellular metabolites secretion (23, 26). Although, previous studies have demonstrated that the enhanced production of bioactive molecules synthesized by bacteria, yeast, filamentous fungi, and plants after ultrasonic, there are no extensive reports on the improved potential of ultrasonication for the productivity of biosurfactants. Our previous studies demonstrated the enhanced effect of ultrasound treatment on the biosurfactant efficiency from *L. plantarum* using the synthetic culture media, which could not be cost-effective. Hence, the present study aims to obtain a low-cost biosurfactant using agro-food wastes while applying ultrasonication treatment in different stages of cell growth to enhance biosurfactant production. Since the composition of growth media can result in variation of cell growth and then biosurfactant structure, the produced biosurfactants using synthetic and waste-based media were structurally compared using Fourier transform infrared spectroscopy (FTIR) and Nuclear magnetic resonance spectroscopy (1H NMR). Antiviral activity of biosurfactant from *L. plantarum* was also tested against Newcastle disease viruses (NDV) LaSota strain. Antiviral activity was measured for biosurfactants derived from both synthetic and waste-based culture media in order to assess their effectiveness and economical potential.

## Materials and methods

### Experimental design and statistical analysis

During the present study, three different experiments were carried out including the evaluation of *L. plantarum* potential to produce biosurfactant using agro-food wastes, selection of an agro-food waste-based medium to study the effect of ultrasound on biosurfactant production, and investigation of antiviral activity of biosurfactant produced in waste-based medium assisted by ultrasound against NDV LaSota strain.

At the beginning, four waste materials (date syrup, sugar cane molasses, rice bran and husk hydrolysate, whey permeate) were selected as fermentation media for the production of *L. plantarum*-derived biosurfactant by changing the amount of each waste described in Table 1. Fermentation was performed in a 250 ml Erlenmeyer flask with 100 ml of the waste materials described in Table 1 at pH 6.5. The flasks containing media were inoculated using 2 ml of the subculture followed by incubation (37°C, 120 rpm). After 48 h of fermentation, surface tension activity was measured to determine biosurfactant activity, as described elsewhere (16, 27) using a tensiometer (Nanometric, Contact angle-101, Iran) according to the Wilhelmy plate method. Data analysis was performed using SPSS v.22.0. Analysis of variance (ANOVA) was carried out to determine significant differences and means were separated using the Tukey test. Statistical significance was defined as a probability value of < 0.05. Accordingly, whey permeate and sugar cane molasses in combination (50:50) (medium of 9) were statistically chosen as the most effective wastes to continue the optimization of biosurfactant production.

**Table 1:** Chemical composition of the substrates used in the present study.

**Substrates**	**Total sugar (g/L)**	**Protein (g/L)**	**Zn (mg/L)**	**Fe (mg/L)**	**Cu (mg/L)**	**Mn (mg/L)**	**Ca (mg/L)**	**Mg (mg/L)**
Date syrup	44	3.2	0.52	0.073	0.09	0.03	75.36	57.2
Sugar cane molasses	56	5	0.46	3.81	0.19	0.02	797.7	19.52
Rice bran and husk hydrolysate	21.5	2.3	0.11	3	0.18	0.005	23.65	42.32
Whey permeate	45.5	2.9	0.05	0.31	0.21	0.29	50.37	38.4

Response surface methodology (RSM, Estat-Ease Design Expert 10.0.7) was employed to optimize some variables responsible for the critical conditions in biosurfactant production. Three variables affecting the biosurfactant production were included yeast extract (1–5 g/L), inoculum size (1–5%), and fermentation time (24–72 h) at three levels by a central composite design (CCD) in 20 runs (**Table 3**), while the experimental response was selected to be surface tension measurement (that depends on biosurfactant concentration). Analysis of Variance (ANOVA) was assessed to determine the effect of different variables on the biosurfactant production. The best conditions were validated and reported as mean ± standard deviation.

To assay the role of different ultrasonication times in the cell growth and biosurfactant production, the flasks including the fermentation media were treated by ultrasound for a given time of 30 min at different cultivation stages throughout the fermentation process (0, 4, 8, and 12 h of fermentation). The obtained results were statistically analyzed (SPSS version 22.0 software) to estimate any significant differences between results. Analysis of variance (ANOVA) and the Tukey test were utilized by the probability value of < 0.05. No ultrasonication was applied for the control sample.

Finally, the antiviral activity of biosurfactants solutions from waste-based medium (BS_1_) and standard MRS broth (BS_2_) at different doses (0.93 to 7.5 mg/ml) were compared against NDV LaSota strain.

### Microorganism and inoculum preparation

*L. plantarum* ATCC 8014 was preserved in de Man Rogosa Sharpe broth (MRS: Merck, Germany), at −80°C freezer. Frozen strains were streaked on MRS agar, and overnight incubated at 37°C. The samples were kept for 2 weeks at 4 °C. A subculture solution was prepared by cultivating a colony to 10 ml of sterile standard MRS broth (4 g/L of yeast extract, 20 g/L of glucose, 10 g/L of peptone, 5 g/L of C_2_H_9_NaO_5_•3H_2_O, 2 g/L of K_2_HPO_4_, 2 g/L of C_6_H_17_N_3_O_7_, 0.2 g/L MgSO_4_•7H_2_O, 0.05 g/L MnSO_4_•4H_2_O and 1 ml of C_24_H_44_O_6_) and overnight incubated at 37°C with shaking at 120 rpm.

### Preparation of agro-food wastes

Sugar cane molasses was provided by the Iranian Sugar Company of Eqlid, Iran. It was prepared as described previously by Rodrigues et al. (16, 28). The solution pH was set at 6.5 and clarified before autoclaving for 15 min at 121°C (16).

Whey permeate was procured from Pegah Dairy Factory-Shiraz and kept at 20°C for later use. It was prepared as 10% suspension and the pH value was adjusted to 4.5. For denaturing the proteins, the solution was heated (121°C, 15 min). To remove the precipitates, the solution was centrifuged (8,000 × g, 10 min at 4°C). Then, the supernatant was modified by setting pH to 6.5 and sterilizing at 121°C for 15 min, which was then utilized as culture media (29).

Dates (*Phoenix dactylifera* var. *Kabkab*) were acquired from a market in Kazeroon City, Fars, Iran. To prepare date syrup a modified method introduced by Moosavi-Nasab et al. (30) was used (30). In short, the low-quality dates were wetted by soaking in distilled water (50°C) for 30 min and then mixed thoroughly. Using a double-layer cheesecloth, the homogenized extract was filtered, and the remaining was rinsed using hot water (85°C). The Brix (total soluble solid) and pH of the resultant syrup were set at to 10 and 6.5, respectively. Then, the date syrup was autoclaved at 121°C for 15 min.

Rice bran and husk were provided from local threshing rice, Kazeroon, Iran. The material was ground in a knife mill until an average particle size of 1 mm in diameter was obtained. The processed substances were maintained at room temperature until experimentation. 20 g of rice bran and husk were properly mixed with 200 ml of distilled water and hydrolyzed using the steam explosion method (at 121°C for 2 h). Then, the steam-exploded hydrolysate was clarified through centrifugation (8,000 × g, 15 min at 4°C) and the pH was adjusted to 6.5 and used as culture media after autoclaving (121°C for 15 min) (31).

All the agro-food wastes were characterized using chemical methodology, kjeldahl and atomic absorption spectroscopy (Shimadzu/AA-67OG, Japan) for total sugar, protein, and minerals analysis (Zn, Fe, Cu, Mn, Ca, and Mg), respectively (18). Moreover, the concentration of lactose in whey permeate and glucose, fructose and sucrose in sugar cane molasses were assessed using high performance liquid chromatography (HPLC) (AZURA/KNAUER, Germany).

### Ultrasonic treatment setup

An ultrasonic bath (300 × 150 × 150 mm) (PARSONIC 7500S, PARS NAHAND ENGG. Co., Tehran, Iran) was used to perform ultrasonication with a fixed frequency of 28 kHz and a power input of 100 W. According to the previous studies, low frequency (lower than 100 kHz) and intensity (lower than 2 W/cm^2^) ultrasonication lead to beneficial effects on microbial cell growth and then metabolite production owing to the cell permeability and mass transfer (32, 33). The 250 ml Erlenmeyer flasks with 100 ml of the culture media were supported in a stainless steel basket 10 mm from the bottom of the ultrasonic bath. In addition, water level was proportionate to the level of the flasks content. The flasks were positioned in the center of bath, and temperature was remained fixed at 25°C. After that, the flasks were placed into the shaker incubator for running the fermentation process (72 h).

### Extraction of cell-bound biosurfactant

To extract the cell-bound biosurfactant, cells harvesting was done through centrifugation (10,000 × g, 5 min, 4°C) followed by washing with sterile distilled water twice, and then suspension in 20 ml of phosphate-buffered saline (PBS:10 mM KH_2_PO_4_/K_2_HPO_4_, and 150 mM NaCl, pH 7.2). Following similar studies, the bacterial suspension was gently shaken (60 rpm) at room temperature for 2 h (Roller Mixer, Pole Ideal Pars, Iran) to collect the cell-bound biosurfactants (27). Afterwards, the cells were then separated through centrifuging (10,000 × g, 5 min, 4°C), and the remaining cell-free suspensions were filtered *via* 0.22 μm pore size filters (CHROMAFIL^®^ Xtra, Germany). The filtrates dialysis was done using Spectra/Por5 dialysis membrane (molecular weight cut off 6,000–8,000 Da, Membrane Dialysis Products, Spectrum, USA), in distilled water at 4°C for 24 h lyophilized, weighed, and then kept at −20°C for further studies (27).

### Determination of critical micelle concentration and surface activity

A tensiometer was used to measure the surface tension value of supernatants (Nanometric, Contact angle-101, Iran) according to the Wilhelmy plate method at room temperature to check the surface activity of the isolated cell-bound biosurfactants suspended in PBS solution as described elsewhere (15). To calibrate the tensiometer, the surface tension of distilled water was employed. After three time measurements, the average of the replicates were calculated. The critical micelle concentration (CMC) refers to the significant concentration of the biosurfactant solution that forms micelles. To obtain the CMC value, the surface tension of diluted biosurfactant solutions in distilled water ranging from 0.1 to 10 mg/mL (g biosurfactant/L solution) were measured and plotted up to a constant value (27).

### Glucose consumption

A commercial reagent (Biorexfars, Iran) including peroxidase (POD) and glucose oxidase (GOD) enzymes was used to measure the glucose concentration. The enzymatic glucose measurement was carried out as the manufacturer procedure at 546 nm wavelength and the value of glucose was determined afterwards.

### Structural characteristics of BS

#### Fourier transform infrared structure identification

The primary functional groups in the cell-bound biosurfactants isolation (freeze-dried biosurfactant) were characterized using a Fourier transform infrared (FTIR) spectroscopy. At the wavenumber ranging from 400 to 4,000 cm^−1^, the IR spectrum of samples was measured using FTIR spectrophotometer at a scanning speed of 2 mm/s (Tensor II, Bruker, Germany).

#### Nuclear magnetic resonance spectroscopy

Freeze dried biosurfactants were dissolved in D_2_O (50 mg/ml) and the respective 1H NMR spectra were recorded at 25°C using a 400 MHz NMR spectrometer (Avance III, Bruker, Germany). Chemical shifts in 1H NMR were measured in ppm in comparison to a solvent shift used as a chemical standard.

### Antiviral assay

#### Biosurfactant preparation

Firstly, 30 mg/ml biosurfactant solutions from waste-based medium (BS_1_) and standard MRS broth (BS_2_) were prepared by dissolving the freeze-dried biosurfactants in PBS. Then the solutions were filtered through sterile membrane filters (pore size, 0.22 μm) and kept in sterile vials at −20°C until further used.

#### In ovo toxicity of biosurfactants solution

To evaluate biosurfactants toxicity on the egg embryo, the concentrations of 30, 15, 7.5, 3.75 and 1.87 mg biosurfactants per ml of PBS solution were prepared. A 100 μL of the prepared concentrations were injected into the allantoic cavities of three 9-day-old chicken embryo eggs, constituting five groups according to the biosurfactants dose. The eggs were incubated in a humidified atmosphere for 48 h at 37°C. After incubation, the size and appearance of chicken embryo were recorded.

#### Virus propagation

The effectiveness of biosurfactants solutions (BS_1_ and BS_2_) at different doses (0.93–7.5 mg/ml) were examined against NDV LaSota strain. The strain was supplied from school of veterinary medicine, Shiraz university, Shiraz, Iran. A mixure of different doses of biosurfactants (100 μL) and virus strains (100 μL) supplemented with antibiotic and antifungal agents were propagated into three 9-day-old chicken embryo eggs and then incubated in a humidified condition at 37°C for 48 h. The eggs were moved to refrigerator at 4°C and then the allantoic fluids were collected and the presence and activity of the virus strains in the allantoic floids of each eggs were examined using haemagglutination (HA) assay (34–36).

#### Determination of median embryo infectious dose (EID50) of NDV LaSota

In this case, seven-fold serial dilution of NDV LaSota strain were prepared as 10^−1^ to 10^−7^. Then, the 30 mg/ml biosurfactants (BS_1_ and BS_2_) were dissolved in PBS solution. After that, the biosurfactant solutions were mixed with equal volume of virus diluents. Nine-day-old embryo chicken eggs labeled as 10^−1^-10^−7^ were inoculated with 0.1 ml/egg of the virus diluents (V) and 0.2 ml/egg of the mixture solutions (VBS_1_ and VBS_2_). Five eggs were inoculated in each dilution. After incubation in humidified condition at 37°C for 48 h, the haemagglutination activity was characterized and EID_50_ and neutralization index were calculated (35, 37–39).

### Ethics approval

This research was approved by the local Ethics Committee of Shiraz University and complies with Shiraz University animal welfare guidelines and policies.

## Results

### Biosurfactant production using agro-industrial wastes

As mentioned, the ingredients of the employed agro-industrial wastes including date syrup, sugar cane molasses, rice bran and husk hydrolysate, and whey permeate were examined. The results showed that the substrates consisted of carbohydrate [total sugar (g/L)], protein (g/L) and minerals [Zn, Fe, Cu, Mn, Ca, and Mg (mg/L)] (Table 1). The results showed that the concentration of fructose, glucose, and sucrose in sugar cane molasses were 10.5, 8.6, and 32.4 g/L, respectively. The concentration of lactose in whey permeate was measured 39.3 g/L. Therefore, the focus is on using low-cost raw materials as the production medium to reduce production costs; however, several wastes have been selected for use as biosurfactant production medium by *L. plantarum* ATCC 8014.

Fifteen waste-based media (Table 2) were subjected to assess the biosurfactant production by *L. plantarum*. According to the results from Table 2, *L. plantarum* ATCC 8014 was able to grow and produce biosurfactant in all the agro-food wastes. It led to reduce the surface tension of PBS solution from 72 to the range of 49.6 to 43.93 mN/m. Surface tension value is the main parameter indicating biosurfactant production. The maximal decrease on the surface tension of media such as water and PBS demonstrates the effectiveness and efficiency of the produced biosurfactant. The various wastes were selected as culture media for producing biosurfactant by *L. plantarum*, which exhibited varying levels of biosurfactant production. Results from the present study displayed surface tension reduction when growing the strain in date syrup and rice bran and husk hydrolysate, which is in accordance with other studies (18, 40, 41). In addition, the biosurfactant production with the medium consisted of 50% of whey permeate and 50% of sugar cane molasses (medium of 9) was significantly high (43.93 ± 0.14 mN/m) in comparison with the other waste materials gained in Table 2 (*p* < 0.05), although use of each (whey permeate and sugar cane molasses) alone showed significantly higher surface tension value. Media and culture conditions i.e., fermentation time, pH, temperature, and nutrient ingredients are known to influence the yield of biosurfactant production. However, this part of the study was conducted to produce biosurfactants from *L. plantarum* ATCC 8,014 grown in 15 waste-based media without any supplementation. Therefore, the screened waste-based medium (50% of whey permeate and 50% of sugar cane molasses) was used for further optimization based on the concentration of yeast extract as nitrogen source, size of the inoculum, and fermentation time in the next part. Consequently, agro-industrial waste was observed as a vital factor, which reduced the cost of biosurfactant production and also served as a source of nutrients for *L. plantarum* ATCC 8014.

**Table 2:** The percentage of waste materials in each run and surface tension activity of *L. plantarum*-derived biosurfactant.

**Run number**	**Date syrup (%)**	**Whey permeate (%)**	**Rice bran and husk hydrolysate (%)**	**Sugar cane molasses (%)**	**Surface tension (mN/m)**
1	100	–	–	–	45.595 ± 0.19^b^
2	–	100	–	–	47.425 ± 0.19^c^
3	–	–	100	–	45.7 ± 0.32^b^
4	–	–	–	100	45.66 ± 0.24^b^
5	50	50	–	–	45.68 ± 0.15^b^
6	50	–	50	–	49.77 ± 0.32^e^
7	50	–	–	50	49.23 ± 0.9^de^
8	–	50	50	–	45.46 ± 0.43^b^
9	–	50	–	50	43.93 ± 0.14^a^
10	–	–	50	50	47.915 ± 0.28^cd^
11	33.3	33.3	33.3	–	47.37 ± 0.38^c^
12	33.3	33.3	–	33.3	46.75 ± 0.35^bc^
13	33.3	–	33.3	33.3	49.6 ± 0.26^e^
14	–	33.3	33.3	33.3	45.67 ± 0.32^b^
15	25	25	25	25	45.77 ± 0.14^b^

a, b, c, d The letters indicate the significant difference between surface tension value of biosurfactants from 15 waste-based media.

### Optimization of the culture condition for biosurfactant production

Our current study has concentrated on the use of agro-industrial wastes to produce *L. plantarum*-derived biosurfactants in a cost-effective manner, as well as the improvement of the sustainability of technical processes. Once the optimum waste-based media consisted of 50% of sugar cane molasses and 50% whey permeate were screened by traditional one factor at a time method, RSM proceeded under CCD to increase the production and the significant factors optimization in biosurfactant production. To this end, three variables were selected namely yeast extract (A); inoculum size (B); and fermentation time (C). The lower/upper bounds of A (g/L), B (% v/v), and C (h) were 1/5, 1/5, and 24/72, respectively. The design expert software (CCD) generated 20 experimental set-ups (Table 3). The response included surface tension value (mN/m), which determined experimentally using specific conditions. The achieved experimental response (surface tension) was fitted to the model. The predicted values of response are listed in Table 3 and as seen, they are highly consistent with the experimental outcomes. Analysis of variance (ANOVA) had verified the model, which was critical to evaluating acceptability and significance as indicated in Table 4. The correlation coefficients were R^2^ > 0.93, which means a high level of similarity of predicted and the experimental values. Table 4 lists the ANOVA results for the independent variables (A, B, and C) and the interactions (AB, BC, AC, A^2^, B^2^, and C^2^). The results of ANOVA analysis revealed that the quadratic model, variables (yeast extract, inoculum size, and fermentation time), and interactions of the variables (AB, BC, A^2^, and C^2^) were statistically significant (*p* < 0.05). In addition, the model was suitable for optimal production of biosurfactant using *L. plantarum* as indicated by the non-significance of the lack of fit test (*p* > 0.05) (Table 4). The model was significant (*p* < 0.0001), and the R^2^ and adjusted R^2^ values were 97.01% and 94.32%, respectively. The use of the RSM to determine optimal parameters has led to an empirical relationship between the test variables and the responses. The quadratic polynomial equation below corresponds best to the data:


$$\begin{array}{l} Y=51.68-2.10A-2.20B-0.75C+0.163AB-4.04\\ \;\times 10^{-3}AC+1.01\times 10^{-1}BC+0.23A^{2}+9.02\times 10^{-2}B^{2}\\ \;+1.06\times 10^{-3}C^{2} \end{array}$$

**Table 3:** Central composite design runs showing actual and predicted variables and the responses.

**Run number**	**A** **Yeast extract (g/L)**	**B** **Inoculum size (%)**	**C** **Fermentation time (h)**	**Responses (Y)** **Surface tension (mN/m)**	
				**Predicted values**	**Actual values**
1	1 (−1)	3 (0)	48 (0)	45.31	45.19
2	5 (+1)	5 (+1)	72 (+1)	46.39	46.21
3	5 (+1)	1 (−1)	72 (+1)	45.42	45.53
4	5 (+1)	3 (0)	48 (0)	43.70	43.42
5	3 (0)	3 (0)	48 (0)	43.57	43.31
6	3 (0)	3 (0)	48 (0)	43.57	44.15
7	3 (0)	3 (0)	48 (0)	43.57	43.55
8	5 (+1)	5 (+1)	24 (−1)	42.47	42.67
9	3 (0)	3 (0)	72 (+1)	45.61	45.86
10	1 (−1)	1 (−1)	72 (+1)	48.74	48.65
11	5 (+1)	1 (−1)	24 (−1)	44.39	44.57
12	1 (−1)	5 (+1)	24 (−1)	42.39	42.4
13	3 (0)	5 (+1)	48 (0)	43.04	43.1
14	3 (0)	1 (−1)	48 (0)	44.82	44.36
15	1 (−1)	5 (+1)	72 (+1)	47.08	47.02
16	3 (0)	3 (0)	48 (0)	43.57	43.36
17	3 (0)	3 (0)	24 (−1)	42.75	42.1
18	3 (0)	3 (0)	48 (0)	43.57	43.95
19	1 (−1)	1 (−1)	24 (−1)	46.93	47.22
20	3 (0)	3 (0)	48 (0)	43.57	43.95

**Table 4:** ANOVA analysis for response surface quadratic model for biosurfactant production in terms of surface tension measurement (mN/m).

**Source**	**Sum of squares**	**DF**	**Mean square**	**F-Value**	***p*-Value**	
Model	**56.54**	9	6.28	36.05	< 0.0001	Significant
A-Yeast extract	6.53	1	6.53	37.47	0.0001	
B-Inoculum size	7.97	1	7.97	45.77	< 0.0001	
C-Fermentation time	20.48	1	20.48	117.52	< 0.0001	
AB	3.42	1	3.42	19.62	0.0013	
B	0.30	1	0.30	1.72	0.2186	
BC	4.16	1	4.16	23.88	0.0006	
A^2^	2.41	1	2.41	13.82	0.0040	
B^2^	0.36	1	0.36	2.06	0.1821	
C^2^	1.03	1	1.03	5.89	0.0356	
Residual	1.74	10	0.17			
Lack of fit	1.13	5	0.23	1.82	0.2626	Not significant
Pure error	0.62	5	0.12			
Cor total	58.28	19				
Std. Dev.	0.42			R-squared	0.9701	
Mean	44.53			Adj R. squared	0.9432	
C.V.%	0.94			Pred R-squared	0.8479	
PRESS	8.87			Adeq precision	21.490	

Source, A meaningful name for the rows. Sum of Squares, Sum of the squared differences between the overall average and the amount of variation explained by that rows source; DF, Degrees of Freedom: The number of estimated parameters used to compute the source's sum of squares; Mean Square, The sum of squares divided by the degrees of freedom. Also called variance. F Value, Test for comparing the source's mean square to the residual mean square. P-Value, Probability of seeing the observed F-value if the null hypothesis is true (there are no factor effects). Small probability values call for rejection of the null hypothesis. The probability equals the integral under the curve of the F-distribution that lies beyond the observed F-value.

where, Y is surface tension (response), A is yeast extract, B is inoculum size, C is fermentation time.

**Figure 1:**
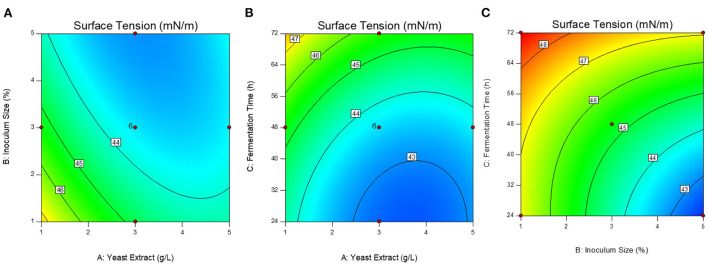
Response surface contour plots showing the interactive effect of variables on the surface tension. **(A)** Yeast extract g/L: inoculum size % (AB); **(B)** Yeast extract g/L: fermentation time h (AC); **(C)** Inoculum size %: fermentation time h (BC).

The optimized conditions were predicted as 2.4 g/L of yeast extract, 4.8 % of inoculum size (v/v), and 26 h of fermentation time. The contour response surface plots indicating the process variables have interactive effects on the response (surface tension) are presented in Figure 1. The concentration of yeast extract: inoculum size (AB) and inoculum size: fermentation time (BC) interactions that induce a significant effect on the process of production (Figures 1A,C). To validate the model, the optimal conditions were applied and the experimental response i.e., surface tension value was obtained as 41.86 ± 0.24 mN/m.

### Effect of ultrasound on fermentation profile and biosurfactant production at optimized conditions

**Figure 2:**
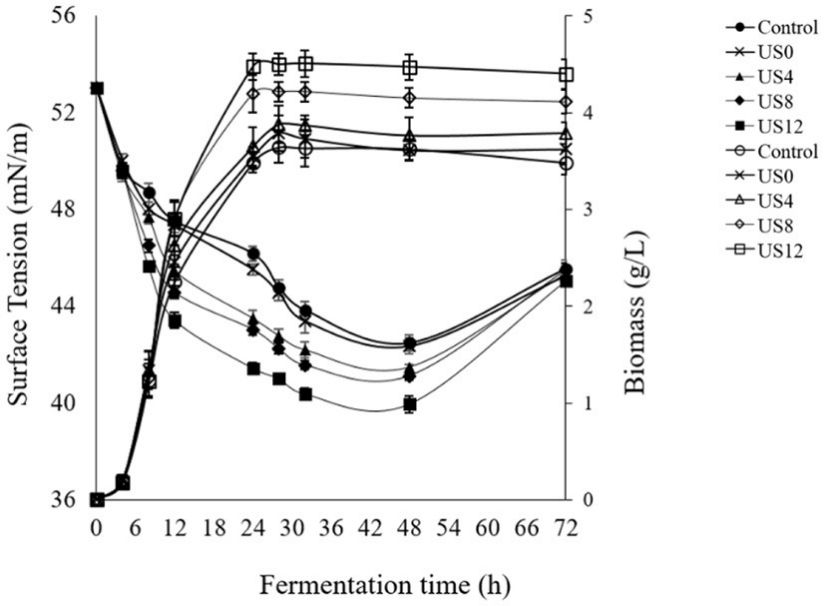
Effect of ultrasound treatment at different growth stages of *L. plantarum* (ATCC 8014) on biomass concentration (g/L) and biosurfactant production [surface tension value (mN/m)]. Filled symbols: surface tension values and hollow symbols: biomass concentration.

**Figure 3:**
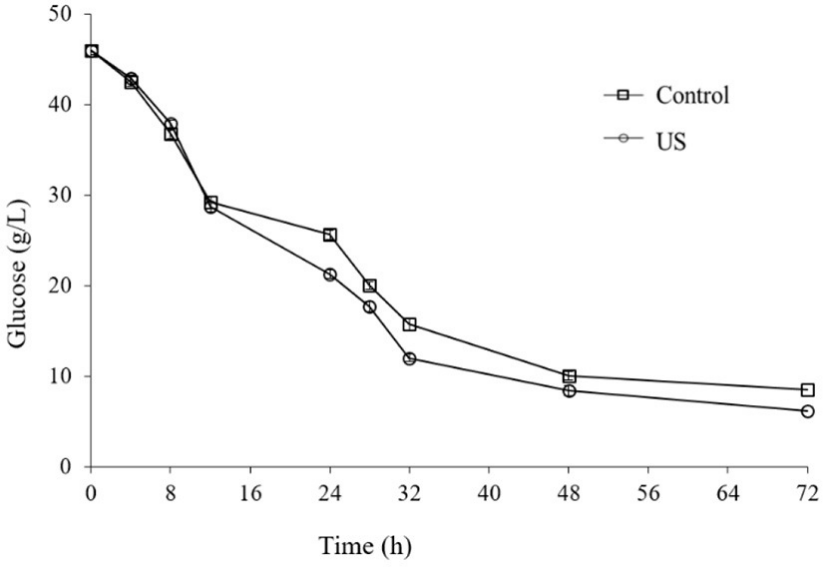
Effect of ultrasound treatment at stage of 12th h of *L. plantarum* (ATCC 8014) growth on glucose consumption (g/L).

Based on previous works by Avhad and Rathod (42) and Maddikeri et al. (21), the current study investigated the influence of ultrasound (28 kHz, 100 W) at various phases of microbial growth to enhance biosurfactant production using agro-industrial wastes leading to a low-cost product. The optimized culture medium and condition from previous Section consisted of sugar cane molasses (50% v/v) and whey permeate (50% v/v) supplemented by 2.4 g/L of yeast extract and cultured by 4.8% of subculture and then 26 h of fermentation time were applied for examination of ultrasonication treatment on the cell growth and production of biosurfactant. Figures 2, 3 show the profiles of fermentation of the treated and non-treated *L. plantarum* ATCC 8014. The bacterial growth and surface activity are described by the growth curves at different stages of the fermentation (i.e. 0th, 4th, 8th, and 12th h) (Figure 2) and glucose consumption at 12th h of fermentation (Figure 3) influenced by ultrasonication with a frequency of 28 kHz for 30 min. The control sample is one that has not been sonicated. According to Figure 2, biosurfactant production is a growth-associated property, since a direct association between bacterial growth and production of biosurfactant (indicated by a decline in the surface tension) is found. There was not any significant differences between the bacterial growth curves when sonication treatment happened at the 0th and 4th h of the fermentation process and the control (without sonication). On the other hand, ultrasonication at the 8th and 12th h of the fermentation significantly affected the bacterial growth. Although no significant differences were obtained between sonication at 8th and 12th, treatment at 12th h of fermentation caused a higher biomass concentration (4.47 ± 0.04 g/L). However, ultrasonic treatment at the 12th h of fermentation was more effective compared to the others (Figure 2). Moreover, biosurfactant production affected by ultrasonication at the 12th h of the fermentation process significantly reduced the surface tension value (39.95 ± 0.35 mN/m) compared to the control (42.49 ± 0.34 mN/m) and samples sonicated at 0th (42.35 ± 0.3 mN/m), 4th (41.46 ± 0.14 mN/m), and 8th (41.10 ± 0.02 mN/m) h of the process after 48 h.

From Figure 2, it demonstrates that there is no significant increase in biomass concentration until 5th h compared to the control, showing the lag phase of the microbial growth, while the surface tension sharply decreased which shows the presence of surface-active agents on the bacterial cells. Furthermore, fermentation including sonicated and non-sonicated fermentation has a stable increase in dry cell mass and surface tension reduction until 48 h. A reduction in surface tension reduction and slightly cell dry mass was observed when the fermentation process was extended to 72 h displaying disintegration of the cells and consequently the cell-bound biosurfactants. Accordingly, sonication at the 12th h of fermentation was significantly more efficient than the others (0th, 4th, and 8th) (*p* < 0.05). However, as is clear in Figure 3, consumption of carbohydrate was significantly affected by ultrasonic treatment of the culture media at the 12th h of fermentation compared to the control. During the first 12 h of fermentation, moderate consumption of glucose occurred (Figure 3). From this time until the completion of the fermentation, the level of glucose consumption increased slightly, while that of exposure to the ultrasound at the 12th h of fermentation increased sharper than non-sonicated fermentation (control).

### Characterization of the synthesized biosurfactant

**Figure 4:**
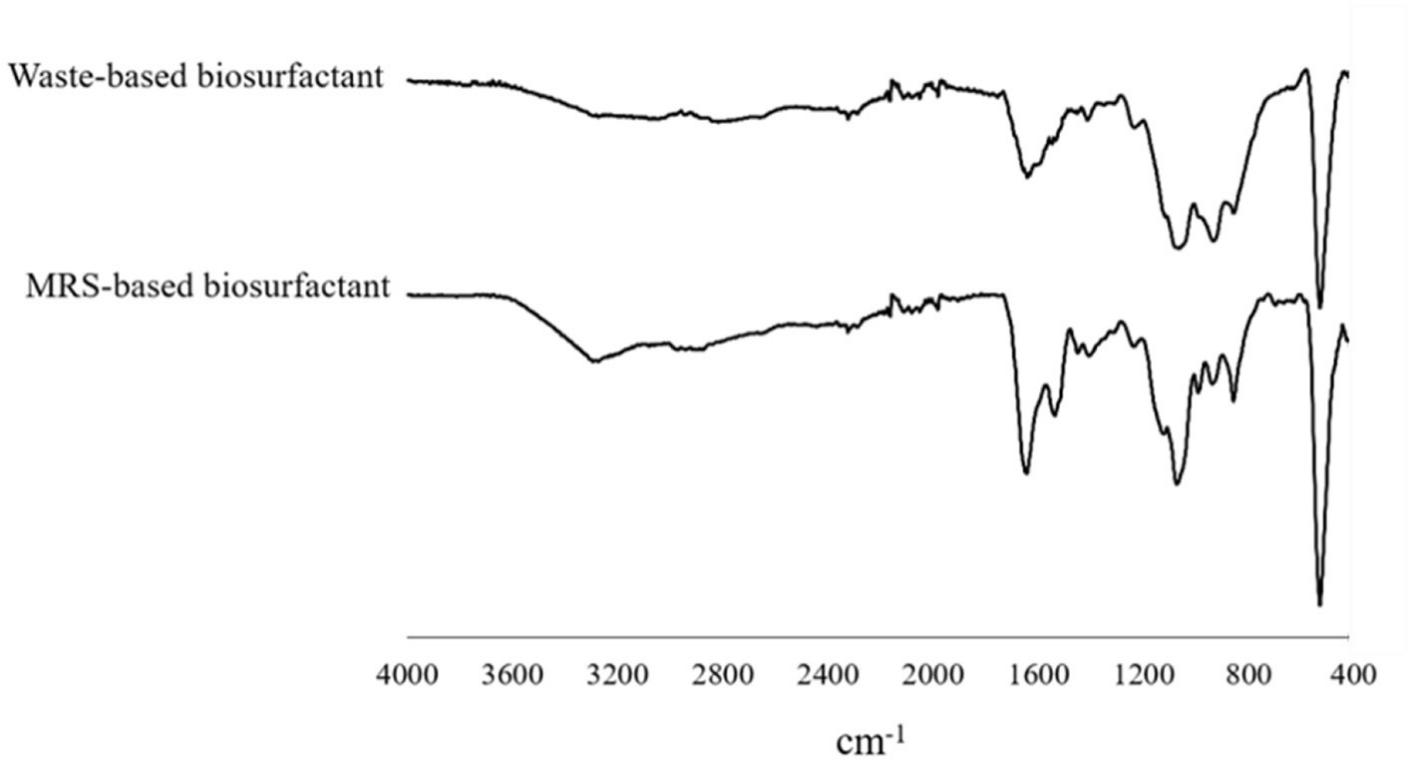
FT-IR spectral analysis of biosurfactant from *L. plantarum* ATCC 8014 grown in MRS and waste-based media.

As shown in our previous study, any alteration was observed in the biosurfactant structure profile influenced by sonication. However, the effect of low-cost substrate on the structure of biosurfactant produced by *L. plantarum* ATCC 8014 compared to the MRS broth was examined here. No changes in the biosurfactant structure profile produced in MRS and waste-based media by *L. plantarum* were determined using FTIR. Thus, as illustrated in Figure 4, there was no definite difference. The determined spectra were relatively similar, representing the inexistence of culture media impression. Therefore, the significant bands in 1,537.79 cm^−1^ and 1,643.98–1,641.4 cm^−1^ range were related to N-H bending vibration in Amides II and C=O stretching of Amids I, respectively (41, 43). The band at 1,073.19 and 1,062.1 cm^−1^ corresponded to the glycoside band vibration of COC in polysaccharide fraction. The IR spectra at the wavenumber of 857.7 cm^−1^ suggested the stretching of the α-isomeric carbon, which was supported by 1H NMR results from the present study. The phosphorus and oxygen (POC) stretching band in aromatic and aliphatic molecules is located within 937.7–935.25 cm^−1^ (4, 41). The 1,450–1,400 cm^−1^ bands were related to CH bending vibration in aliphatic chains as similarly reported by Bakhshi et al. (41). The small peaks in the 1,236–1,235 cm^−1^ range are presumably related to vibrations of COC in esters. Other studies have found C–O (ether bond) (4).

**Figure 5:**
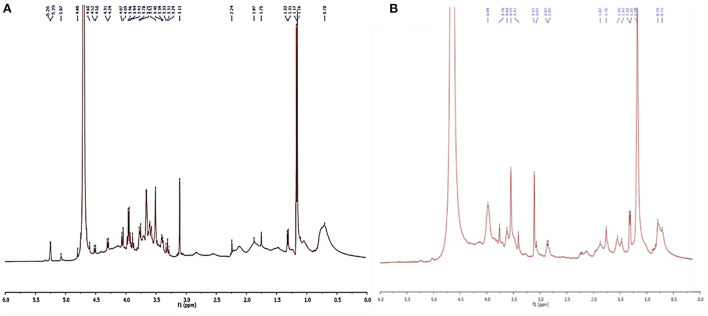
NMR analysis of biosurfatant produced by *L. plantarum* ATCC 8014. **(A)** Biosurfactant from waste-based medium; **(B)** biosurfactant from MRS broth.

1H NMR analysis of the *L. plantarum*-derived biosurfactants from MRS and waste-based media dissolved in D_2_O is also displayed in Figure 5. From the figure (Figures 5A,B), it can be observed that typical culture media almost influenced the biosurfactant composition from *L. plantarum*. In the 1H NMR spectrum (Figure 5A), chemical shifts at 5.26 and 5.07 ppm were explained by the anomeric proton of the α-1, 4-linked D-glucopyranose, and α-1, 6 D-glucopyranose, respectively, while it was not observed for biosurfactant from MRS culture broth (Figure 5B). Moreover, several chemical shifts from 5.07 to 3.11 ppm (Figure 5A) and 3.98 to 3.07 ppm (Figure 5B) were assigned to the olefinic protons of double bonds and also indicating the presence of fractions of polysaccharides (4, 44). The range between 3 and 0.7 ppm corresponds to the presence of glucan–protein structure considering the N–CH_3_ and N–H groups (41). Signals corresponding to the aliphatic chains (CH_3_ and CH_2_) are also observed in the 1H NMR spectra at 1.87 to 0.7 ppm range (4) in the biosurfactant produced in MRS and waste-based media.

### Determination of critical micelle concentration

The freeze-dried biosurfactants obtained from low-cost substrates with concentrations between 0.1 and 10 mg/ml were dissolved in PBS, (pH 7.0). Thus, a significant decline of surface tension of PBS from 72 ± 0.26 mN/m reached its minimum (39.23 ± 0.38 mN/m) at the concentration of 1.75 mg/mL, corresponding to the CMC value and no more reduction was observed in surface tension measurement after adding to the biosurfactant concentration.

### Evaluation of antiviral activity

#### In ovo toxicity and antiviral activity of biosurfactants from waste-based and standard media

**Figure 6:**
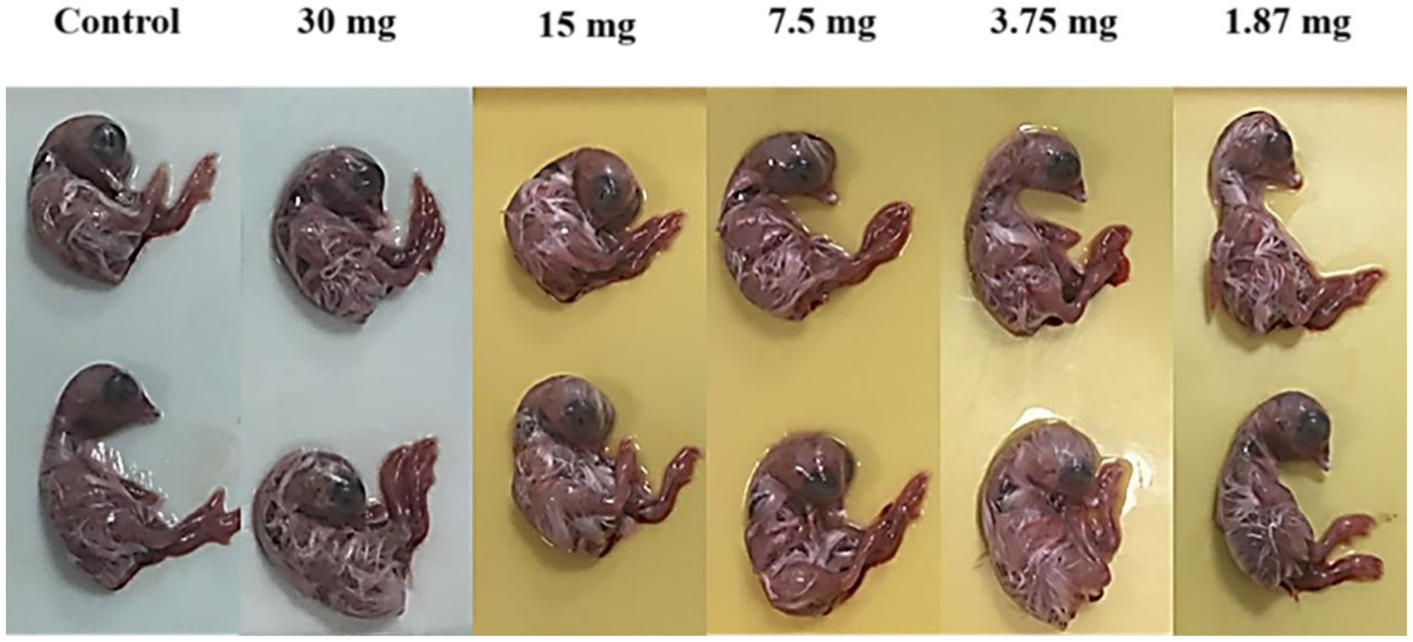
Effect of biosurfactants (at different doses for 48 h) on size and aspect of chicken embryos.

In the present study, we examined the comparison between *L. plantarum*-derived biosurfactants from synthetic and waste-based media as antiviral agents against NDV LaSota strain. Both biosurfactants (BS_1_ and BS_2_) produced in the present study were safe and non-toxic for embryos at different concentrations after 48 h incubation. Toxicity of biosurfactants (BS_1_ and BS_2_) were examined 48 h after inoculation of the 9-day-old embryonated eggs with different concentrations of the agents (30, 15, 7.5, 3.75 and 1.87 mg/ml). No changes in size and appearance, and growth inhibition effects were observed, and embryos growth looked very normal. Results were similar to the untreated eggs or control samples indicating non-toxicity effect of both biosurfactants solutions on embryos even in the high concentrations (30 mg/ml) (Figure 6).

Antiviral activity of BS_1_ and BS_2_ at different doses (0.93 to 7.5 mg/ml) against NDV LaSota strain were examined and the results were tested using HA assay. As can be obtained from Table 5, the activity of BS_1_ and BS_2_ as determined by HA activity in allantoic fluids indicated the significant enhanced antiviral activity against NDV LaSota by rising in biosurfactants concentration from 0.93 to 7.5 mg/ml. The concentration of 7.5 mg/ml and 3.75 mg/ml had almost 4 HA titer for both BS_1_ and BS_2_, but less concentrations (1.87 mg/ml and 0.93 mg/ml) showed the higher HA titer. Accordingly, further study was performed on the efficiency of higher doses of antiviral agents against NDV LaSota strain to compare BS_1_ and BS_2_ effectiveness, since no reports on antiviral activity of biosurfactant derived from lactobacilli against NDV strains have been published. The concentration of NDV LaSota untreated and treated with BS_1_ and BS_2_ in a suspension is expressed as the infectivity titers (EID_50_%). Analysis of infectivity titres resulted from titration are calculated by following equation:


$$\begin{array}{l} \text{Dilution}=\frac{\%\text{ Mortality at dilution just above }50\%\;-\;50\%}{\%\text{ Mortality at dilution just above }50\%\;-\;\%\text{ Mortality at dilution just below }50\%} \end{array}$$

**Table 5:** Haemagglutination activity of NDV LaSota, vNDV, Influenza H_9_N_2_ strains treated by BS_1_ and BS_2_ solutions in embryonated chicken embryos.

**Treatment**	**Concentration (mg/ml)**	**HA titer**
		**NDV LaSota**
BS_1_	7.5	4
	3.75	4
	1.87	7
	0.93	7
BS_2_	7.5	4
	3.75	4
	1.87	7
	0.93	7
Virus control	–	8
BS1 Control	3.75	Negative
BS2 Control	3.73	Negative

As can be observed from Table 6, for virus strain, %mortality just above 50% obtained at dilution of 10–6 equal to %86 and %mortality just below 50% obtained at dilution of 10–7 equal to %33. According to the equation, dilution will be 0.68 and the dilution producing the infection rate above 50% is dilution of 10–6.68 and as a result, EID_50_ unit for NDV LaSota suspension is 106.68/100 μL. Therefore, the EID_50_ unit for treated strain with BS_1_ and BS_2_ were calculated in the same way and were obtained 104.5/100 μL and 104.83/100 μl, respectively. Then, log NI for BS_1_ is 2.18 by which NI value will be 151.36 and log NI for BS_2_ is 1.85 and NI value will be equal to 70.8. As can be deduced from the results EID_50_ unit for treated virus strain with low-cost biosurfactant (BS_1_) is significantly higher than that of treated with biosurfactant from synthesized biosurfactant (BS_2_) and they potentially inactivated NDV LaSota strain at the concentrations of 7.5 mg/ml and 3.75 mg/ml.

**Table 6:** Median percent embryo infectious dose of NDV LaSota strain treated by BS_1_ and BS_2_ solutions.

**NDV LaSota dilution**	**Injected eggs**			**Positive HA**			**Negative HA**			**Mortality (%)**		
	**Virus**	**VBS_1_**	**VBS_2_**	**Virus**	**VBS_1_**	**VBS_2_**	**Virus**	**VBS_1_**	**VBS_2_**	**Virus**	**VBS1**	**VBS2**
10^−1^	5	5	5	5	5	5	0	0	0	100	100	100
10^−2^	5	5	5	5	5	5	0	0	0	100	100	100
10^−3^	5	5	5	5	5	5	0	0	0	100	100	100
10^−4^	5	5	5	5	5	5	0	0	0	100	100	100
10^−5^	5	5	5	5	0	2	0	5	3	100	0	40
10^−6^	5	5	5	4	0	0	1	5	5	86	0	0
10^−7^	5	5	5	2	0	0	3	5	5	33	0	0

## Discussion

Date syrup, sugar cane molasses, rice bran and husk hydrolysate, and whey permeate were expected the adequate substrates for lactic acid bacteria growth, since the lactic acid bacteria are extremely fastidious organisms and need complex nutrients to grow (18). The production of biosurfactants is highly dependent upon the type and quantity of ingredients present in the culture media. Selecting the right sources of nitrogen and carbon or other nutrients is major step in designing a productive and cost-effective biosurfactant production process (45). For instance, (41) reported the rice bran hydrolysate as the only substrate between different agricultural by-products for biosurfactant production by *L. plantarum* PTCC 1896 resulting in a significant emulsification activity. Another study by Ghasemi et al. (18) was attained a significant surface tension reduction (41.97 %) from *L. rhamnosus* by using low-quality date syrup (18). The high biosurfactant production with sugar cane molasses and whey permeate is attributed to the presence of valuable sugar, protein, minerals, vitamins, and organic acids for the fermentation process (45, 46). To our knowledge, few studies were conducted using whey permeate and sugar cane molasses used as the source of carbon for the production of biosurfactant from *L. plantarum*. Rodrigues et al. (16, 28) used cheese whey and molasses instead of MRS synthetic media and M17 broth, for the production of biosurfactants by *Lactococcus lactis* and *Streptococcus thermophilus*, achieving a 1.2–1.5 times increase in biosurfactant concentration for each gram of dry cell biomass and a 60–80% reduction in medium preparation costs (16). Mouafo et al. (44) found that sugar cane molasses caused the highest emulsification index due to the presence of proteins and minerals as well as sugar, which can enhance the growth rate of *L. plantarum* G88, *L. delbrueckii* N2, and *L. cellobiosus* TM1 leading to biosurfactant production (44). Another study by (46) reported the maximized biosurfactant production using *Bacillus licheniformis* and *B. subtilis* when cultured in molasses for 72 h at 45°C under shaking conditions. Also, Shi et al. (47) achieved the maximum rhamnolipid production when applied waste cooking oil as the sole carbon source for *Pseudomonas aeruginosa* M4 growth (47).

Different researchers have been investigated that low-intensity ultrasonication at sub-lethal levels enhances microbial growth rate by stimulating or creating cavitation process. Moreover, the microbial growth phase has an important role in the stimulation effect of sonication in which the cells are exposed to ultrasound. It has been reported that sonication treatment in the lag phase causes premature cell harm, slowing cell growth. On the other hand, in the stationary phase, sonication is not able to alter the growth profile and synthesis of the product. Once cell growth enters the stationary phase, the growing intensity of *L. plantarum* will weaken. A possible reason for this effect could be related to the accumulation of unwanted products or the loss of nutrients that inhibit cell growth (26). Furthermore, sonication in the growth phase can improve productivity by increasing the penetration of the cell membrane and subsequently substrate uptake (21, 42). Thereupon, it can be attributed to the closer resonance frequency of the cell membrane to the low-frequency ultrasound (i.e., 28 kHz frequency), which occurs maximum entering the ultrasound energy to the cell, generating the biophysical effects which accelerate the cell growth and thereby accumulate the cell-bound biosurfactant (48). The present results are in accordance with our previous study applying a modified MRS culture medium for biosurfactant production by *L. plantarum* ATCC 8014 with some exceptions (3). A probable reason for this occurrence is found to lose cell bunches, increase cell permeability inducing gas-liquid and solid-liquid mass transfer, which enhances substrate uptake, improving cell generation, and subsequently higher dry cell mass and metabolites production (i.e., biosurfactants) (49, 50). Moreover, sonication treatment enhances the release of internal enzymes (like β-galactosidase) from the cells, enhancing lactose hydrolysis, and then accelerating the fermentation process (51). Another relevant reason for increasing substrate consumption is related to shatter the substrate ingredients to form smaller constituents, therefore increasing the uptake of nutrient and oxygen *via* cell membranes (52). In addition, an essential role of ultrasonication in the stimulation of carbohydrate consumption and organic acid production would be attributed to the electroporation as increases the cell penetration accelerating the transfer of nutrients through the cell membrane (53), which is one of the mechanisms explained in the literature for stimulation of fermentative processes related to the ultrasound (51). The literature review showed three studies conducted by (21), (49), and our group (3, 54) employing an ultrasonic treatment to induce biosurfactant production. Maddikeri et al. (21) reported similar results by applying ultrasonication (40 kHz, 600 W, and 10 min) during the exponential phase of *Starmerella bombicola* fermentation. The outcome of sophorolipid production was found to be 24.7 g/L using used cooking oil as the production medium (21). Sheikh et al. (49) reported increased rhamnolipid production by *P. stutzeri* using ultrasonication (150 W) with 6 min of irradiation, and 42.5% duty cycle (49). However, there has never been a report of production of biosurfactant utilizing *Lactobacillus* strains driven by ultrasound and agro-industrial wastes. Therefore, according to the hypothesis reported by previous studies (32, 33, 50, 54) the facilitating role of ultrasound also was found from the present study. As shown in Figure 2, the biosurfactant production was improved by enhancing the biomass concentration, displaying cell-associated production of biosurfactant by *L. plantarum* ATCC 8014 as previously reported studies (40, 55). Therefore, the factors contributing to the higher biomass production also accumulate cell-bound biosurfactants.

The minimal biosurfactant concentration needed the amphipathic molecules to form the micelles and the surface tension value minimized was considered as the CMC value of the synthesized biosurfactant (46, 56). The CMC value indicates the efficiency of biosurfactants and is the crucial factor for practical objects. However, a sufficient surfactant causes a lower CMC point since a very small amount of surfactant is required to lower the surface tension value, as biosurfactants synthesized by *B. subtilis* R1 and *B. licheniformis* K51 could decrease the surface tension of water (72 mN/m) to 33 mN/m (46). Moreover, the CMC value in the current study represents a comparable value to previous CMC values from other biosurfactants derived from lactic acid bacteria and the synthetic SDS equal to 1.8–2.9 mg/mL with the surface tension equal to 37 mN/m (8, 28). Presently, the investigation of biosurfactants antiviral activity are mostly limited to surfactin and sophorolipid biosurfactants synthesized by *Bacillus* and *Candida*, respectively. To the best of our knowledge, this is the first study considering *Lactobacillus*-derived biosurfactants against NDV LaSota strains. In the present study, a comparison between antivairal activity of biosurfactants from synthetic and waste-based media against NDV LaSota strain was examined. Both synthesized biosurfactants potentially inactivated NDV LaSota strain at the concentrations of 7.5 mg/ml and 3.75 mg/ml which is in accordance to the previously report describing the potential of lipopeptide biosurfactants synthesized from *B. cereus* against NDV strain which showed the antiviral activity at the concentration of 10 mg/ml. Lipopeptide biosurfactants have displayed lytic membrane properties which cause antimicrobial activity against different microbial cells (9). Different chemical groups constitute biosurfactants molecules having potential application in various areas especially known as antimicrobial agents (57). Biosurfactants are able to influence the microbial morphogenesis and membrane structure of the host cells due to the amphiphilic structure with active hydrophobic and hydrophilic domain (58). Results exhibited that microbial glycolipoprotein extracted from synthetic and waste-based media both directly inhibited envelope NDV LaSota strain at the concentration comparable to the other reports by (37). Although both bioproducts (BS_1_ and BS_2_) characterized the similar structures based on FTIR and NMR spectrums, biosurfactants extracted from waste-based medium was significantly more effective than that of from synthetic broth. It can be associated to the active groups generated by bacterium strain in the media while some studies have been explained the effect of diverse medium components on the production of microbial biosurfactants (55). Moreover, the antiviral efficacy of biosurfactants against a variety of enveloped viral strains causes by the formation of ion channel on the capsid and lipid coating of viruses membrane which results in the loss of proteins involved in adsorption/penetration processes and then the prevention of viral membrane fusion. A recent study with similar results displayed the potential of lipopeptide mixtures against NDV and Porcine epidemic diarrhea viruses indicating biosurfactants as new antiviral agents (59). Although the antiviral biosurfactants have not still applied in clinical cases, pre-clinical examinations have demonstrated their potentially applications in pharmacology and therapy sectors. The one important reason for not clinical using these substances is associated to the economical point of view into commercial biosurfactant production. Nevertheless, cost-effective and large-scale production of biosurfactants are fundamental approaches to develop widespread use of these microbial surface-active agents in disease prevalence such as COVID-19 pandemic (60).

## Conclusion

Sugar cane molasses and whey permeate were screened as the basic media and culture condition were optimized using RSM central composite design. Ultrasonication applying at 12th h of the fermentation process was selected as optimal ultrasonic time, by which the surface tension was lowered (39.95 ± 0.35 mN/m) rather than the control (42.49 ± 0.34 mN/m). Structural characterization of biosurfactants by FTIR and 1H NMR spectroscopy revealed that no significant differences were found between biosurfactants from both culture media and they were identified as glycolipoprotein structures. However, the low-cost production, structural and functional characterizations as well as GRAS (generally recognized as safe) status of the bacteria, highlight economically the potential applicability of the *L. plantarum*-derived biosurfactant postbiotic in the different processes, particularly in pharmaceutical and food industries. Although the antiviral biosurfactants postbiotics have not still applied in clinical cases, pre-clinical examinations have demonstrated their potential applications in pharmacology and therapy sectors. The one important reason for not clinical using these substances is associated to the economical point of view into commercial biosurfactant production. Nevertheless, cost-effective and large-scale production of biosurfactants are fundamental approaches to develop widespread use of these microbial surface-active agents in disease prevalence such as COVID-19 pandemic.

## Data availability statement

The original contributions presented in the study are included in the article/supplementary material, further inquiries can be directed to the corresponding authors.

## Ethics statement

This research was approved by the local Ethics Committee of Shiraz University and complies with Shiraz University animal welfare guidelines and policies. Written informed consent was obtained from the owners for the participation of their animals in this study.

## Author contributions

AB carried out the experiments and wrote the manuscript. All authors edited the manuscript, designed, and managed the experiments. All authors contributed to the article and approved the submitted version.

## Funding

This work was supported by the Vice Chancellor for Research and Technology, Shiraz University of Medical Sciences [Grant Number 23715].

## Conflict of interest

The authors declare that the research was conducted in the absence of any commercial or financial relationships that could be construed as a potential conflict of interest.

## Publisher's note

All claims expressed in this article are solely those of the authors and do not necessarily represent those of their affiliated organizations, or those of the publisher, the editors and the reviewers. Any product that may be evaluated in this article, or claim that may be made by its manufacturer, is not guaranteed or endorsed by the publisher.
